# Bedside visualisation tool for prediction of deviation from intended dosage in multi-infusion therapy

**DOI:** 10.1177/11297298221146327

**Published:** 2023-01-27

**Authors:** Robin JF Gevers, Maurits K Konings, Agnes van den Hoogen, Annemoon MDE Timmerman

**Affiliations:** 1 Department of Medical Technology and Clinical Physics, University Medical Centre Utrecht, GA Utrecht, The Netherlands; 2 Department of Neonatology, Wilhelmina Children’s Hospital, University Medical Centre Utrecht, Utrecht, The Netherlands

**Keywords:** Multi-infusion, bedside visualisation tool, dosing errors, push-out effect, Poiseuille flow, mechanical compliance, neonatal intensive care unit

## Abstract

**Background:**

In multi-infusion therapy, multiple infusion pumps are connected to one single vascular access point. Interaction between pressure changes from different pumps may result in temporary dosing errors, which can be very harmful to the patient. It is known that these dosing errors occur. However, clinicians tend to find it hard to estimate the order of magnitude of these errors.

**Methods:**

This research uses an existing mathematical model to create a bedside prediction tool that is able to provide clinicians with the dosing errors that will occur after flow rate changes in multi-infusion therapy. A panel of clinicians, consisting of both nurses and doctors, was formed, and, in order to assess the level of knowledge about dosing errors in multi-infusion, the panel was presented with four medication schedules in which a syringe exchange or change in flow rate took place. The panel was asked to predict the resulting dosing errors.

**Results:**

A prediction tool was developed that describes a two pump multi-infusion system and predicts dosing errors resulting from changing the flow rate at one pump. 44% of the panel members wrongly predicted the impact of changing the set flow of liquid A on the flow of liquid B that reaches the patient. Nobody was able to correctly predict the dosing deviation if a very small catheter was used. After the prediction tool was shown, the clinicians indicated they had a improved understanding of what deviations to expect and that the tool would be useful in understanding multi-infusion dosing errors.

**Conclusions:**

Using the predictive tool to visualise the deviations from the set flow rate is an effective method to allow clinicians to gain insight in dosing errors in multi-infusion therapy. This knowledge can be used to better anticipate future dosing errors in clinical situations.

## Introduction

### Multi-infusion therapy

In intravenous infusion therapy, fluids are administered directly into the blood stream. Creating a vascular access point, introduces risks, such as catheter-related bloodstream infections (CRBSI) and catheter-related thrombosis.^1–4^ Fletcher indicates that CRBSI has a mortality rate of up to 25% and CRBSI significantly increases hospital length of stay and overall treatment cost.^1^ It is therefore desirable to create as few access points as possible, which can be achieved by connecting multiple infusion pumps to the same access point.^5^

A vascular access device (VAD, e.g. catheter) can be used to create a vascular access site to administer fluids to the patient. Gallieni et al.^6^ describe the different types of VADs and when each type is to be applied.

In drug delivery it can be of great importance to know exactly how much of a certain drug enters the bloodstream of the patient at any given time. van der Eijk et al.^7^ demonstrated that variability of the flow rates at relatively low flow rates can lead to problems in intravenous drug therapy particularly for very young children. Subsequently, Ethgen et al.^8^ demonstrated that dosing errors caused by multi-infusion also pose a threat to adult patients. It is known that the set flow rate on the pumps is not always exactly the same as the flow rate of the liquid entering the patient.

It should be noted that not all drugs are compatible, characteristics such as pH, osmolarity, viscosity can be very different for different infused solutions.^9,10^ A multi-lumen catheter can be used to administer multiple drugs at once through one catheter, while preventing the fluids from mixing with each other before entering the blood stream. However, multi-lumen catheters are often limited to two or three lumen, while even more different fluids needs to administered simultaneously in many clinical situations.

### Causes of deviations from intended medication dose rates in multi-infusion

Furthermore, it is known that the pharmacological contents of the fluid entering the patient at a given point in time, is not equal to the pharmacological contents in other parts of the infusion catheter in most cases. Three distinct factors can be identified that give rise to deviations from the intended medication dose rates in multi-infusion ((i)−(iii), see below)^11,12,17^:

- Push-out effect^13^: The mixing of the fluids in a multi-infusion situation happens at a certain distance from the access point. The volume after the mixing point and before the vascular access point is generally referred to as ‘the dead volume’. The dead volume contains both fluid A and B. If the flow rate of fluid A is changed, the dead volume is pushed out with the new flow rate (new total flow rate of fluid *A* + fluid B), whereas the mixing ratio of the fluids inside the dead volume is still the mixing ratio of the old situation. This results in temporarily administering a different dose then was planned. Only after the dead volume has been pushed out entirely, the dose rates entering the patient correspond to the set flow rates once again. The dosing error resulting from the push-out effect can be limited by reducing the shared volume of fluid A and fluid B, however an error resulting from the dead volume will remain present.
- Poiseuille flow profile^14^: Clinical infusion setups typically have laminar flow patterns, so there is no recirculation or turbulent flow. Infusion lines and catheters can be considered as cylindrical tubes. Within a completely laminar flow in a cylindrical tube, the velocity of the fluid at any given point depends on the distance between that point and the central longitudinal axis of the cylindrical tube. The velocity of the fluid reaches its maximum value at the central longitudinal axis of the tube and is lowest near the wall of the tube. This is called a Poiseuille flow profile, and it only occurs in a laminar flow, as there is no mixing in the radial direction. The flow rate set on the pump equals the average flow rate of the fluid. The maximum flow velocity can be derived from the Hagen-Poiseuille law, which states that the maximum velocity inside the tube equals two times the average velocity inside the tube.
- Mechanical Compliance: Lastly, the mechanical compliance of the causes deviations from the set flow rate.^5,15,16^ Part of the infusion set-up is made of compressible material (most notably, the air-filled rubber plunger in many syringes). If the set flow rate of the pump is increased, the pressure applied to the plunger inside the syringe pump will increase as well. If the flow rate is lowered, the pressure will be lowered as well. In the latter case the compressible (‘compliant’) material will expand as the pressure applied to it is now lower. The expanded material displaces the liquid, thus causing a temporary excess of liquid entering the patient, that is, a dosing error with respect to the instantaneous change in set flow rate.

Decreasing the diameter of a catheter will increase the resistance of the catheter dramatically, since the resistance of a catheter is proportional to d^(−4) in which d is the diameter of the catheter. The combination of high resistance of thin catheters with the compliance of the material in the syringes causes especially counter-intuitive deviations from the set flow rate if a change in flow rate is applied. If the flow in pump A is increased in a two-pump system containing pump A and pump B, pump A starts exerting a higher pressure on the syringe plunger to increase the flow. As the resistance of the catheter is high, quite a lot of pressure is required to obtain the higher flow. The plungers of the syringes in both pump A and B will be compressed in this way. The new pressure applied by pump A initially presses the fluid (partially) back into the tubing coming from pump B. At first, the previously discussed push-out causes a overdosing of fluid B. However, directly after this initial overdosing, an underdosing arrives at the patient due to liquid B being pushed back by the new pressure. This constitutes a highly complex and often counter-intuitive behaviour of the dosing errors.

Anti-syphon valves or non-return valves are able to prevent free flow or backflow, however both are not able to prevent the dosing errors resulting from the three effects that were just described.

The aim of this study was to develop a bedside prediction tool that will predict deviations from the intended dosage in multi-infusion systems and that can be used to explain these deviations to clinicians. Furthermore, the study aims to provide an indication of the level of knowledge of nurses and doctors on dosing errors in multi-infusion situations.

## Method

### Design of the study

For this study, a tool was created to predict dosing errors in multi-infusion systems. Furthermore, a panel of clinicians has been assembled. The members of this panel were presented with four hypothetical multi-infusion cases that resemble clinical situations, for which they are required to indicate the dosing errors they would expect. Afterwards, the results from the prediction tool (developed in our research lab, consisting of a small laptop computer on which the specific software of the prediction tool was running) were presented to the panel, and the panel was asked to evaluate the added value of the prediction tool.

### Software used

The original model^17^ that was used for this prediction tool was written in Mathematica [version 10.3, Wolfram Research inc., Champaign, Illinois, USA]. In this research a combination of Matlab and Labview [version 20.0.1, National Instruments, Austin, Texas, USA] has been used. In Labview, a MathScript Module was used to incorporate the Matlab code. The Mathematica code of the original model was rewritten into Matlab code. Labview was used to create an interactive interface.

### Model behind the prediction tool

The mathematical method used for the prediction tool in this paper is the analytical model created by Konings et al.^17^ As the model is thoroughly explained by Konings et al. only a brief overview of the used model is provided here. The model uses the characteristics of the components used in the multi infusion setup. The method uses the Z-transform to model the contents of the catheter. From this, explicit expressions have been derived automatically by Mathematica. Konings et al. examine the limiting cases and in-vitro measurements were performed to test the validity of the results produced by the model. As these validations have been performed previously, there was no necessity to replicate the validation in this research.

### Simulated setup

The model enables prediction of dosing errors, as induced by a change in the set flow rate of one of the pumps. The input information required are the physical parameters of the system, that is, the mechanical compliance, resistance and the dimensions of all elements. Additionally, the set flow rates (before and after the change in flow rate) must also be provided. As the prediction tool requires the compliance, resistance and dimensions of all elements, it is able to work with any component of which these parameters are known. For the hypothetical examples in this paper the used materials are shown in Table 1

**Table 1.** Used materials in simulations of cases.

Material	Description
Syringe pumps	B Braun Perfusor Space syringe pumps
Syringes	B.Braun Omnifix 50 mL syringes
Tubing	Tubing with a length of 1 m connecting the syringe to the mixing point
Mixing point	B Braun Discofix C 3-gang Manifold
Vascular access device	Two different catheters: The Argon Careflow Single Lumen (7 Fr) and the Vygon Premicath (1 Fr)

### Panel of clinicians

To verify the utility of the prediction tool and to indicate the extent to which clinicians are familiar with the concept that changing the flow rate at one of the pumps will influence the flow from the other pumps, a panel of clinicians has been convened. The panel of clinicians consisted of 32 members and included both doctors and nurses that all worked at the Wilhelmina Children’s Hospital and were working at the Neonatal Intensive Care Unit at the time of this research. They were therefore familiar with both IV management and multi-infusion activities on a daily basis in their clinical practice.

**Figure 1.**
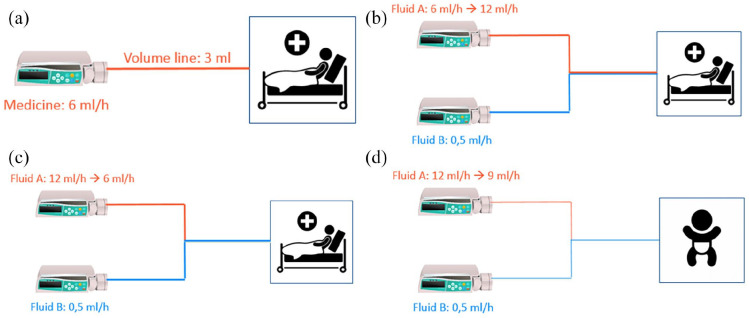
Setups of the (hypothetical) cases. (a) Case 1 shows a simple system of a single syringe pump that is connected to a catheter and flows into the patient. A new syringe filled with a different medicine is then put into the pump that was previously used for another substance. The volume of the tubing and the set flow rate are both given to the members of the panel. The question asked is: how long will it take for the first bit of the medicine to reach the patient? (b)+(c) In case 2 & 3 the described system has two syringe pumps. The tubing comes together at a mixing point and enter the patient through a 7 Fr catheter. Pump A has a much higher flow than pump B. The syringe in pump B is filled with critical medication. The flow of pump A is respectively increased and lowered in case 2 and 3. The panel is asked to predict what will happen to the flow reaching the patient of the medicine that is in the syringe in pump B. (d) The setup of case 4 is very similar to the setup of the second and third case. The difference to previous setups is that this setup utilises a very small catheter of only 1 Fr. The panel is again asked to predict how changes to the flow at pump A, will impact the flow of the critical medicine from pump B entering the patient.

When consulting the panel of clinicians, four hypothetical situations (‘cases’) were presented and the clinicians were asked to predict the resulting dosing error in these cases. The members of the panel were asked to predict changes to flow rates and document their answers individually, without discussing the cases with other members of the panel. The four cases each described a scenario in which a change was applied to the set flow rate on a pump. In Figure 1 the four cases are described.

When the panel members had documented their answers to the questions posed in the four cases, the prediction tool, developed by way of this research, was used to demonstrate predicted outcomes.

### Data handling

In this research no patient data has been used. The data acquisition from the panel of clinicians was performed in such a way that their privacy was secured. Alongside the predictions for the cases, only the profession was provided by the members of the panel.

## Results

A prediction tool has been developed, and four situations (cases) have been analysed, and the results from the panel of clinicians (*N* = 32) have been described.

### Prediction tool

**Figure 2.**
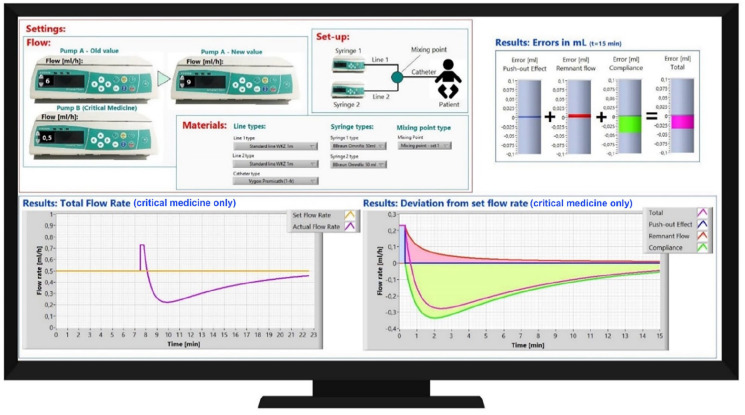
Impression of the predictive tool. In the top-left the two-pump system is described. In this example, the set flowrate of pump A is changed. In ‘Materials’ the syringes and mixing points that are used can be selected from the drop-down menus. The impact of changing the set flowrate on pump A on the resulting flow of the substance in pump B at the vascular access point is presented in the different figures. In the top-right the total dosing errors is shown. The bottom-left figure shows the set flowrate (yellow) and the actual flowrate (purple) of the substance in pump B. The bottom-right figure shows the contribution of different factors to the deviation.

An important part of this research was the development of the prediction tool. In Figure 2 the screen of the prediction tool (consisting of a small laptop computer with a screen on which the specific software of the prediction tool was running) is shown.

### Case analysis

**Figure 3.**
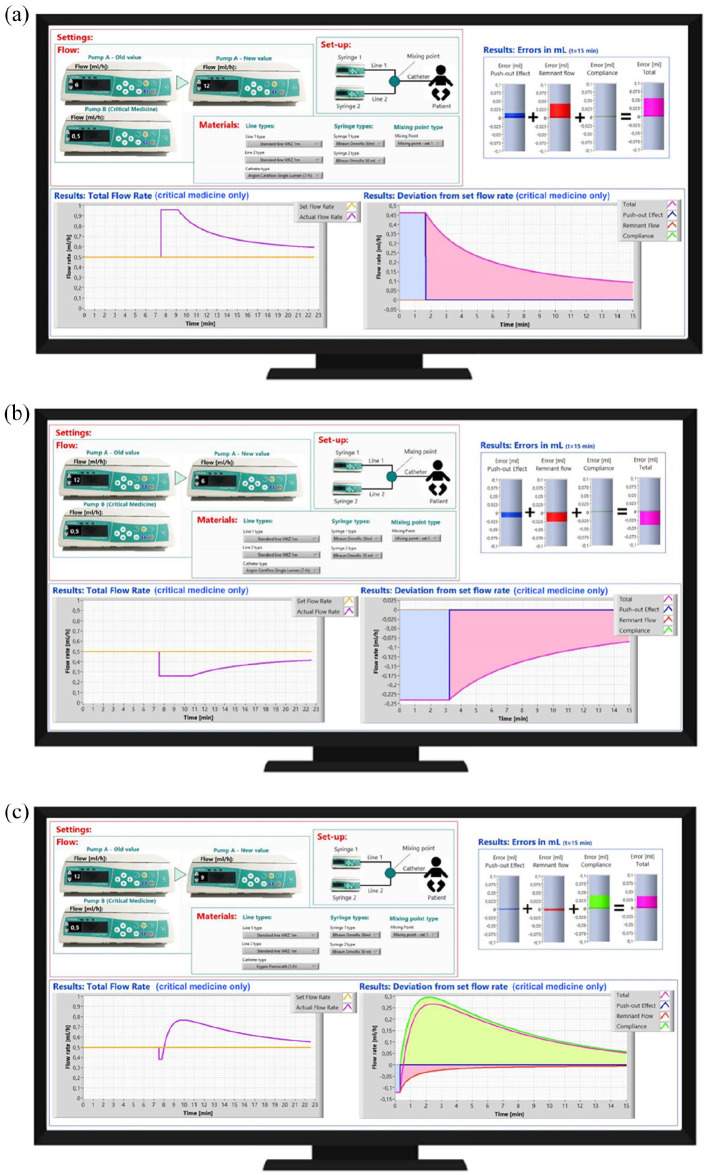
The displays shown in subfigures (a–c) show the prediction of the tool for respectively case 2, 3 and 4. In case 2 (Figure 3(a)) the dosing error is caused by the push-out effect and the Poiseuille flow. When the flow on pump A was increased, a temporary increase in dosing of fluid B occurred. Similarly, a decrease in flow of pump A resulted in a temporary underdosing of fluid B. Case 3 (Figure 3(b)) is very similar to this, however now the effect is opposite to that of case 2 as the flow on pump A has now been reduced. In case 4 (Figure 3(c)) a very small, and therefore high resistance, catheter is used. Now the effect of the error caused by the compliance has a large contribution to the total error, in the way it is also described in the final part of the introduction.

To explain the first case to panel of clinicians, the model was not required. The concept of Poiseuille flow that was explained in the introduction is key in this case. In a Poiseuille flow the first bit of the fluid reaches the end point twice as fast as the average time required by the fluid. The average time required can easily be calculated by dividing the volume of the infusion line by the velocity. This results in a time of 3 mL/(6 mL/h) = 0.5 h. The average time therefor is 30 min and the time it takes for the first bit of the fluid to reach the patient is only 15 min. For the second, third and fourth cases, the model is indicates what happens when flow rates are changed at one pump. The prediction provided by the prediction tool for these cases is shown and explained in Figure 3.

### Panel of clinicians

The panel of clinicians, among them both doctors as well as nurses, consisted of 32 participants. All participants were asked to estimate what would happen in the four cases described in the previous chapter.

**Figure 4.**
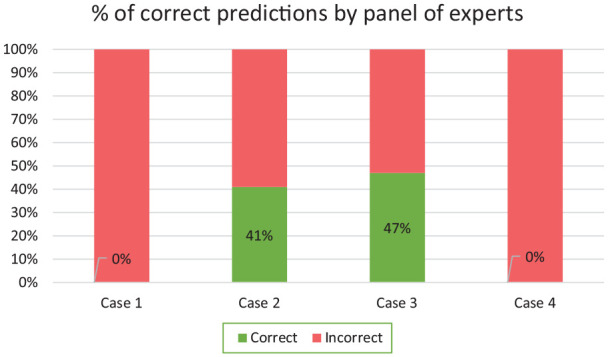
Percentages of correct predictions from the panel of clinicians (experts).

With regard to the first case, none of the participants were able to correctly predict the time it took for the new fluid to reach the patient See Figure 4. When estimating after how much time the first bit of a new fluid would reach the patient, most of the participant used the average flow rate to calculate this, which is also the velocity which is set on the pump. However, the correct answer in this case was twice as small, as a Poiseuille flow profile causes the maximum speed (in the radial centre) to be twice as big as the average velocity.

In the second and third cases, respectively 41% and 47% of our panel of clinicians wrongly predicted what effect changing the flow of fluid A would have on the flow rate of fluid B entering the patient. Many of those who made inaccurate predictions expected the flow rate of fluid B reaching the patient to remain stable even as the set flow rate on pump A was changed. 25% of the participating doctors wrongly predicted the outcome of cases 2 and 3, and 50% of the nurses failed to provide the correct answer.

As a relatively large catheter (7 Fr) for the adult patient in both case 2 and 3 was used, the influence of the compliance of the material of the system did not cause noticeable changes in the flow rate of fluid B.

However, in the case of a thin catheter (1 Fr) having a high resistance, the panel found it especially hard to estimate the deviation caused by the compliance of the material. With regard to case 4, none of the participants (0%) correctly predicted the change in flow rate of liquid B caused by the compliance of the material. There was a common failure to recognise that the compliance of the material causes a greater flow deviation than that caused by the initial push-out effect. When the correct answer was provided, members of the panel indicated that this deviation is counter-intuitive.

## Discussion

We have developed a prediction tool that describes a two pump multi-infusion system and the resulting dosing errors after changing the flow rate at one of the pumps. As has been shown in the results, clinicians find it hard to estimate dosing errors in the four presented cases. Respectively 0%, 41%, 47% and 0% were able to correctly predict the dosing errors in these four cases. The deviations caused by changing the set flow rate using high-resistance catheters was seen as counter-intuitive by the panel. After being presented with the results from the prediction tool, the clinicians indicated that their insight and understanding of dosing errors had improved.

A further future development of the tool may be to apply it to more different situations. As it stands now, the tool is only suited for a two pump system, whereas the model behind the tool does not restrict the number of pumps simulated. Furthermore, in order to improve the generalisability of this research, a larger, more diverse group could be gathered to act as the panel of clinicians. Expanding the group size and including more hospitals and/or more different professions might be interesting. The goals set for the research in this paper, however, were set in such a way that the answer could be provided with the group chosen in this paper.

Another source of dosing errors that the prediction tool could be expanded with is changing the height of infusion pumps with respect to the patient. It has been shown previously that this can also be the cause of a significant dosing error.^18^

Furthermore, in case of a syringe exchange, the pressure drops to nearly zero for a short time, and it has been shown that this leads to dosing errors as well.^19,20^ The tool can be expanded to be able to also predict the consequences of a syringe exchange.

The tool might also be used by a manufacturer to model what dosing errors will occur when their system is used. For instance, the tool could be used to show that a new type of syringe is less prone to multi-infusion dosing errors than current syringes.

When the prediction tool was used to show the panel of clinicians what dosing errors would actually occur in the different cases, a specific question that was asked multiple times was how the clinicians could prevent the dosing error from happening. Knowing that the dosing error will occur is important, but it would be even better to not have the dosing error at all. The underlying mathematical model^13^ could be used by the manufacturer of the pump to create such settings that the dosing error due to multi-infusion characteristics of the system could be mitigated. Also, if the mathematical model would by implemented by the manufacturer, it could provide a warning to the user that a dosing error might occur. Using a control system that incorporates the modelled dosing error would be a valuable addition, as is suggested in other research.^14,21–23^
